# Recurrent Chylothorax: A Rare Complication of Acute Necrotizing Pancreatitis With Necrosectomy and Stent Placement for Pseudocyst

**DOI:** 10.7759/cureus.104318

**Published:** 2026-02-26

**Authors:** Ma. Angela Baquiran, Biruk Amare, Rajvi Patel, Anwar Dudekula, Narayan Dharel

**Affiliations:** 1 Internal Medicine, Mary Washington Healthcare, Fredericksburg, USA; 2 Internal Medicine, Gastroenterology, Edward Via College of Osteopathic Medicine (VCOM), Blacksburg, USA; 3 Division of Gastroenterology and Hepatology, Mary Washington Healthcare, Fredericksburg, USA

**Keywords:** chylothorax, cisterna chyli, gastroenterology, general internal medicine, internal medicine, internal medicine and gastroentrology, necrosectomy, necrotizing pancreatitis, pancreatitis, recurrent chylothorax

## Abstract

Chylothorax is an abnormal collection of chyle in the pleural space that requires prompt intervention. This case report describes a 47-year-old female who presented with recurrent chylothorax as a complication of necrotizing pancreatitis. She was initially evaluated for acute respiratory distress. Chest radiograph showed a large right-sided pleural effusion requiring multiple thoracenteses followed by chest tube placement for symptomatic relief. The patient had a history of acute necrotizing pancreatitis status post cystogastrostomy with axios stent placement and necrosectomy for pseudocyst three years before her presentation. This report highlights inflammatory changes in the pancreas leading to chyle leakage in the pleural cavity. To our knowledge, there are only a few reported cases in the literature. Diagnosis, evaluation, and management explained.

## Introduction

Chylothorax is a rare but potentially serious condition defined by the accumulation of lipid-rich lymphatic fluid in the pleural space, typically from disruption of the thoracic duct or lymphatic drainage pathways [1]. It is most associated with trauma, malignancy, or thoracic surgery, but less commonly, it can arise from inflammatory or obstructive processes such as pancreatitis [1]. We present a case involving a 47-year-old patient with a medical history notable for necrotizing pancreatitis who initially presented with respiratory distress and was noted to develop recurrent right-sided chylothorax. This case suggests a pancreatic-lymphatic connection, where lymphatic disruption due to pancreatic inflammation or necrosis may result in chyle leakage into the pleural cavity. Evaluation of chylothorax includes chest radiograph, thoracic ultrasound, computed tomography of the chest, magnetic resonance imaging of the chest, conventional lymphangiography, or nuclear lymphoscintigraphy [2]. Treatment is guided by the underlying cause and may begin with conservative approaches like dietary modification or drainage.

## Case presentation

A 47-year-old female with a history of hyperlipidemia, hypertension, type II diabetes mellitus, and hypothyroidism was initially hospitalized for necrotizing pancreatitis secondary to cholelithiasis. Her condition progressed with the formation of pancreatic fluid collections, leading to readmission for obstructive symptoms. She underwent transgastric cystogastrostomy with Axios stent placement, draining 2.5 L of fluid. Shortly after, she was readmitted for recurring pancreatitis and underwent endoscopic necrosectomy. Follow-up imaging showed improvement in pseudocyst size and symptoms, and she was referred for elective cholecystectomy. 

**Figure 1:**
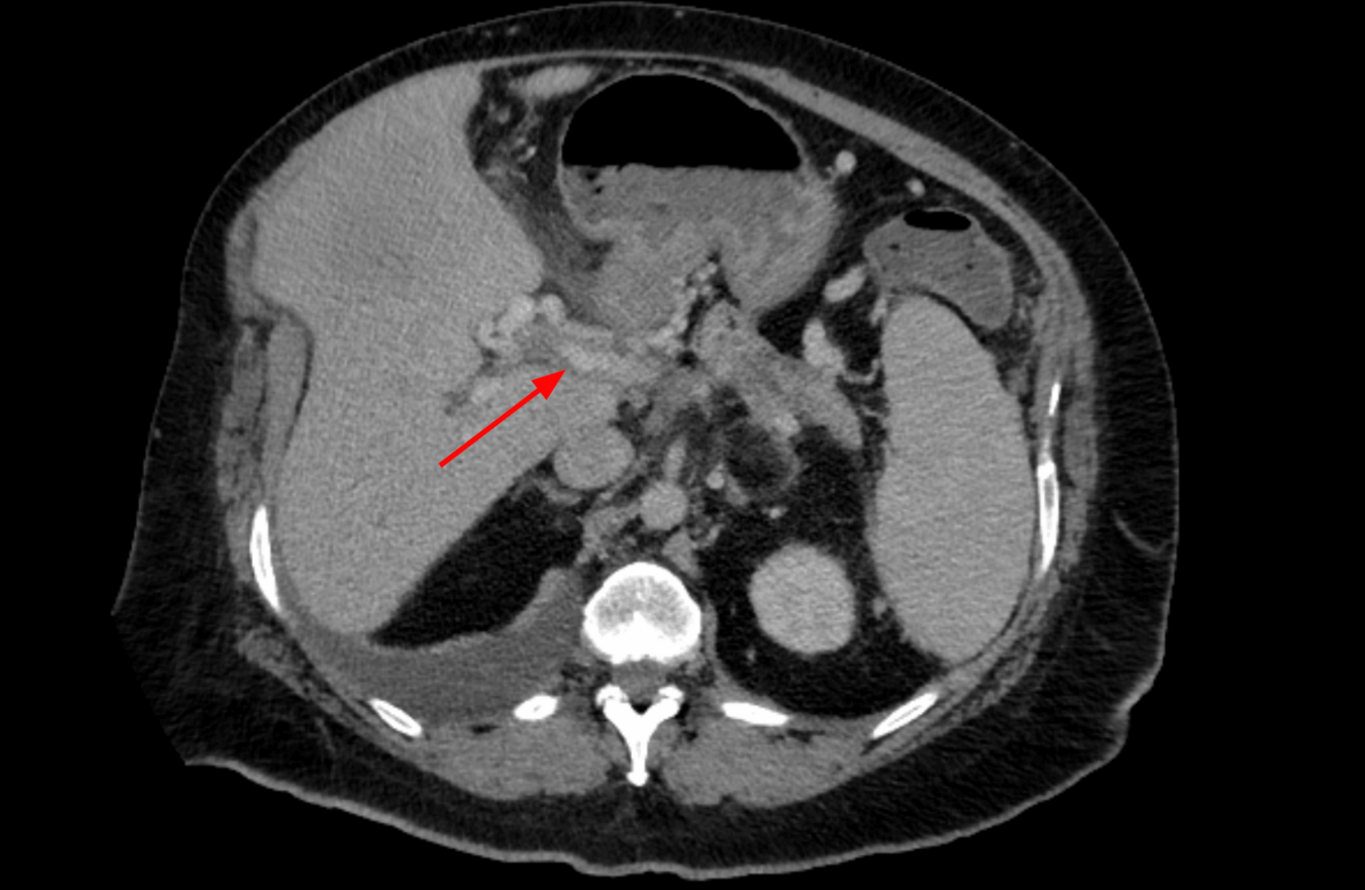
CT abdomen pelvis showing chronic inflammatory changes of the pancreas. Chronic inflammatory changes of the pancreas. CT: computed tomography

**Figure 2:**
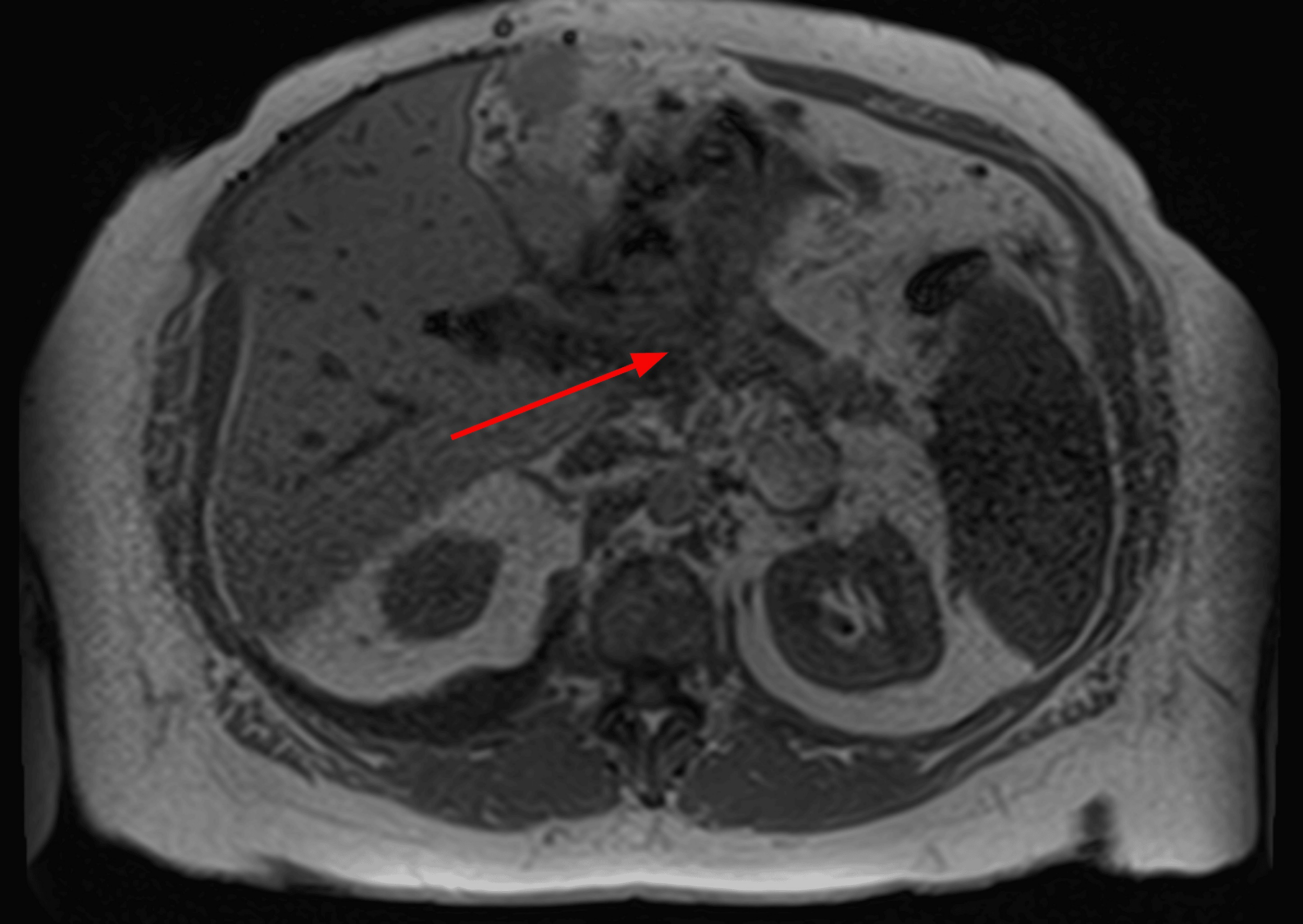
MRI of the abdomen and MRCP showing findings of sequelae of the patient's chronic history of pancreatitis. Chronic inflammatory changes of the pancreas. MRCP: Magnetic resonance cholangiopancreatography

Three years later, the patient presented with acute respiratory failure and was diagnosed with community-acquired pneumonia complicated by right-sided pleural effusion, which appeared chylous on drainage. Fluid analysis was transudative with negative cytology for malignancy. CT abdomen/pelvis (Figure 1) and MRI abdomen MRCP (Figure 2) showed chronic inflammatory changes in the pancreas. Her symptoms resolved after undergoing thoracentesis with a total of 1.5 L of chylous pleural fluid. Fluid study showed 5% neutrophils, 25% lymphocytes, a pH of 7.46, glucose 167, albumin 1.0, protein 2.0, LDH 100, and amylase 37. She was then stable and deemed fit for discharge.

**Figure 3:**
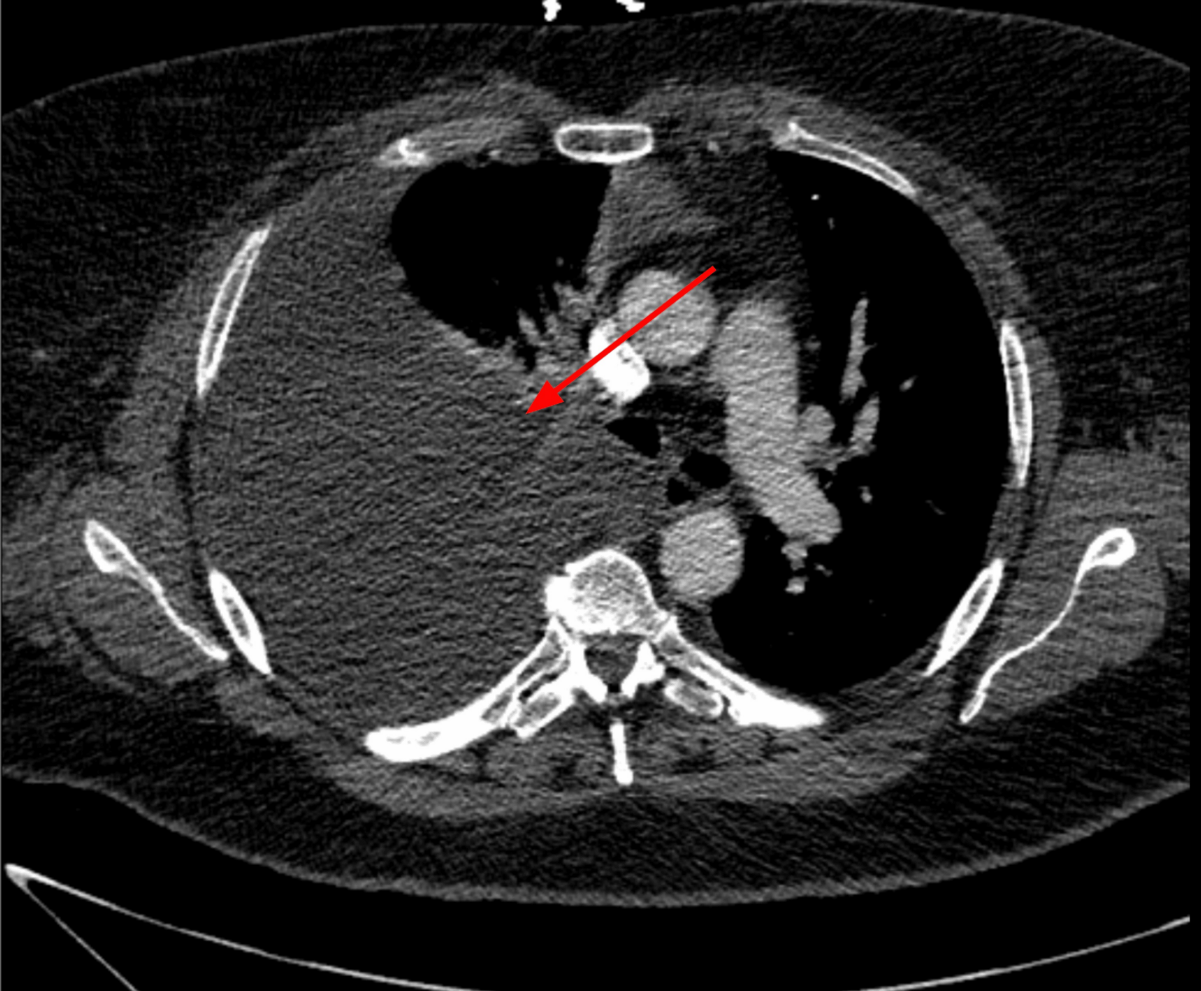
CT chest angio showing a large right pleural effusion. Large right pleural effusion.

She presented to the emergency department with dyspnea and recurrent right pleural effusion several weeks later (Figure 3). Gastroenterology and cardiothoracic surgery were consulted. A right chest tube was placed. Chylous fluid recurrence raised concerns for a possible lymphatic leak below the diaphragm. She was planned for referral to a tertiary care hospital for lipoidal lymphoscintigraphy to evaluate lymphatic leak; however, transfer was delayed due to bed unavailability. She was discharged in stable condition on a fat-free diet. 

**Figure 4:**
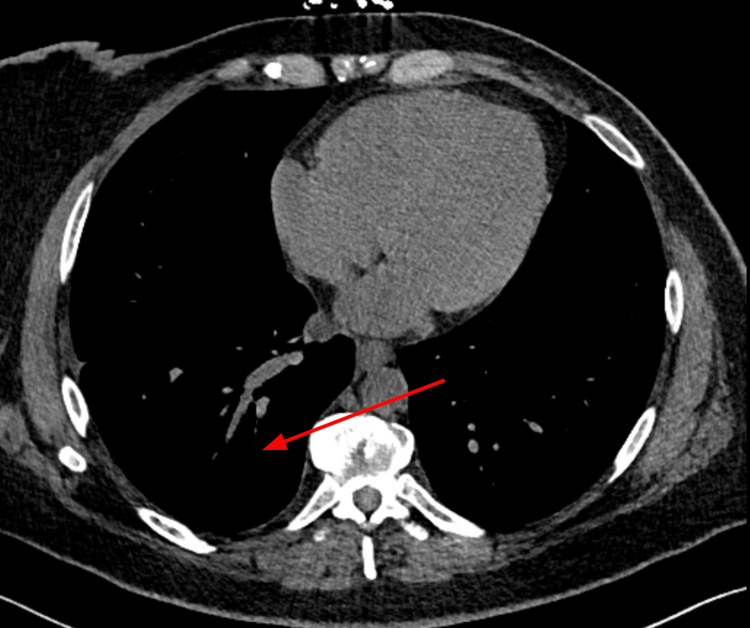
CT chest showing resolution of right pleural effusion. Resolution of right pleural effusion in comparison to Figure 3.

At the follow-up pulmonology office visit, a CT of the chest (Figure 4) was obtained, which revealed significant resolution of effusion with only trace residual fluid. She was cleared to undergo an elective EGD and an MRI of the abdomen to follow up on the sequelae of chronic pancreatitis in 12 weeks. Pending the follow-up MRI results, a referral to a tertiary care center for lipoidal lymphoscintigraphy for chyle leak management is to be considered.

## Discussion

Chyle leaks can present as chylothorax or chylous ascites. The small intestine is an essential site for chyle production due to lipid absorption and secretion into the mesenteric lymphatic system. Nonetheless, either can cause increased risk of morbidity and mortality. Mortality associated with chyle leak complications is up to 70% due to limited knowledge and options for management [3]. Continuous progression of chyle leaks results in protein losses that may progress to malnutrition, weight loss, and further complications such as steatorrhea, edema, and tetany, which is why it is also essential to understand the anatomy of the leak and co-management with nutritional services.

The cisterna chyli is a dilated sac-like structure located in the retroperitoneal space, typically at the level of the L1-L2 vertebrae, just anterior to the vertebral column and posterior to the abdominal aorta. It is situated to the right of the midline, often near the right crus of the diaphragm. The cisterna chyli serves as a confluence for lymphatic vessels, receiving lymph from the lower limbs, pelvis, and abdomen via the lumbar and intestinal lymphatic trunks. It marks the origin of the thoracic duct, which ascends to drain lymph into the systemic circulation at the junction of the left subclavian and internal jugular veins [4]. Its precise location can vary slightly among individuals, and it may be absent or replaced by a lymphatic plexus in some cases [5]. 

Incidence of post-pancreatitis chylothorax is often asymptomatic and is not usually detected. Chylothorax following pancreatitis is a rare complication, with limited data on its precise incidence due to its infrequent occurrence [6]. It typically arises from disruption of the thoracic duct or its tributaries, often secondary to severe acute pancreatitis or chronic pancreatitis, causing pancreatic pseudocyst formation or fibrosis. The leakage of chyle into the pleural space results in chylothorax, which may be unilateral, although most are right-sided, or bilateral. Case reports show that post-pancreatitis chylothorax is more likely in patients with extensive pancreatic inflammation, pseudocyst rupture, or surgical interventions like pancreaticoduodenectomy. Our patient underwent EUS-guided cystgastrostomy with Axios stent placement and subsequent necrosectomy. It is not well established if cystgastrostomy and necrosectomy can lead to chylothorax, and further studies are needed. While the exact incidence rates are not well established, studies also indicate that lymphatic complications, including chylothorax, occur in less than 1% of pancreatitis cases, with even fewer specifically linked to chylothorax [6].

Often, patients with chylothorax who do not require urgent invasive interventions would benefit from pleural drainage with the assistance of a chest tube to address symptom management. With our patient’s presentation of dyspnea due to her recurrent chylothorax, she underwent two thoracenteses, leading to eventual chest tube insertion. Due to the rare limitation of necrotizing pancreatitis-induced chylothorax, it is essential to identify proper management based on the patient’s age, comorbidities, functional status, preference, and rate of re-accumulation.

The role of somatostatin and octreotide has been used for adjunctive therapies with chest tubes and bowel rest in patients with low-output chyle leaks (<1 L/day) [7]. This has often been shown in decreasing indications for surgical repair. Somatostatin and octreotide inhibit gastric, biliary, and pancreatic secretion, which leads to a decrease in fluid volume produced by the thoracic duct [8]. Optimal dosing for both octreotide and somatostatin has varied across the literature; however, there have been studies wherein somatostatin was given by intravenous infusion at 6 mg/day for two weeks or by subcutaneous injection at 50 micrograms every eight hours; octreotide was given in subcutaneous doses of 50 to 200 micrograms every eight hours for 2 to 14 days [8]. Side effects include skin flushing, bradycardia, transient hypothyroidism, and abnormal LFTs [9]. In our patient's case, the patient did not receive either somatostatin or octreotide due to her high-output chyle leak.

Early consultation with in-house dietitians also plays an essential role in chylothorax management. Patients should start on a high-protein and low-fat diet, as it can decrease lipid absorption from the gut and therefore decrease chyle production and eventual re-accumulation [10]. Long-chain triglycerides should be avoided to further decrease conversion into monoglycerides and free fatty acids. Oral nutrition is preferred over the parenteral route of nutrition; however, IV fat emulsions may play a role in patients who are at higher risk of essential fatty acid deficiency. Consumption of fat may gradually increase as symptoms improve. Medium-chain triglycerides can be introduced once chylous output has decreased [10]. 

## Conclusions

Common causes of chylothorax include congenital malformation, tumors, and inflammatory systemic diseases such as sarcoidosis and amyloidosis. However, it is not often seen as a rare complication in patients with a medical history of pancreatitis. It is essential to have a good understanding of the lymphatic anatomy for a low threshold of identifying, diagnosing, and treating chylothorax from a gastrointestinal perspective. 
